# Contour segmentation of the intima, media, and adventitia layers in intracoronary OCT images: application to fully automatic detection of healthy wall regions

**DOI:** 10.1007/s11548-017-1657-7

**Published:** 2017-08-11

**Authors:** Guillaume Zahnd, Ayla Hoogendoorn, Nicolas Combaret, Antonios Karanasos, Emilie Péry, Laurent Sarry, Pascal Motreff, Wiro Niessen, Evelyn Regar, Gijs van Soest, Frank Gijsen, Theo van Walsum

**Affiliations:** 1 Biomedical Imaging Group Rotterdam, Department of Radiology & Nuclear Medicine and Department of Medical Informatics, Erasmus MC, Rotterdam, The Netherlands; 2 Department of Biomedical Engineering, Thorax Center, Erasmus MC, Rotterdam, The Netherlands; 3 Image Science for Interventional Techniques Laboratory, Université Clermont Auvergne, Université d’Auvergne, CNRS, UMR 6284, Clermont-Ferrand, France; 4 Department of Cardiology, Gabriel-Montpied Hospital, Clermont-Ferrand University Hospital, Clermont-Ferrand, France; 5 Department of Interventional Cardiology, Thorax Center, Erasmus MC, Rotterdam, The Netherlands

**Keywords:** Optical coherence tomography, Coronary artery, Contour segmentation, Machine learning

## Abstract

**Purpose:**

Quantitative and automatic analysis of intracoronary optical coherence tomography images is useful and time-saving to assess cardiovascular risk in the clinical arena.

**Methods:**

First, the interfaces of the intima, media, and adventitia layers are segmented, by means of an original front propagation scheme, running in a 4D multi-parametric space, to simultaneously extract three non-crossing contours in the initial cross-sectional image. Second, information resulting from the tentative contours is exploited by a machine learning approach to identify healthy and diseased regions of the arterial wall. The framework is fully automatic.

**Results:**

The method was applied to 40 patients from two different medical centers. The framework was trained on 140 images and validated on 260 other images. For the contour segmentation method, the average segmentation errors were $29 \pm 46~\upmu \text {m}$ for the intima–media interface, $30 \pm 50~\upmu \text {m}$ for the media–adventitia interface, and $50 \pm 64~\upmu \text {m}$ for the adventitia–periadventitia interface. The classification method demonstrated a good accuracy, with a median Dice coefficient equal to 0.93 and an interquartile range of (0.78–0.98).

**Conclusion:**

The proposed framework demonstrated promising offline performances and could potentially be translated into a reliable tool for various clinical applications, such as quantification of tissue layer thickness and global summarization of healthy regions in entire pullbacks.

## Introduction

Optical coherence tomography (OCT) is an imaging modality that enables vascular tissues to be visualized in vivo at a near-histology resolution. Intravascular OCT can provide high-resolution cross-sectional images of the coronary artery and is widely used to assess coronary atherosclerosis [23]. Therefore, OCT is a major asset in clinical applications related to cardiovascular imaging.

However, visual interpretation as well as manual analysis of OCT images suffers from two major drawbacks: the procedure is cumbersome and time-consuming, as well as subject to variability between different analysts. To cope with this issue, a number of methods have recently been proposed to (semi-)automatically analyze OCT images. Stent strut apposition, coverage and re-endothelialization were assessed by means of strut shadow detection [10], active contours [12], and peak intensity location [19]. Contour segmentation methods have been put forward to quantify fibrous cap thickness [25, 28]. Tissue-type characterization was also investigated, by exploiting the backscattering coefficient [26], the attenuation coefficient [8, 21], and image texture [1]. An approach based on matched filtering and hysteresis thresholding was proposed to detect calcified plaques [24]. Another study could successfully classify four tissue types (lipid, fibrous, calcium, and mixed tissues) using local binary patterns and gray level co-occurrence matrices [20]. Recently, a method has been introduced to quantify plaque burden from plaque-free wall measurements [11].

Identification of healthy wall regions is relevant for a great number applications, such as automatic quantification of the extent of coronary artery disease. Nevertheless, automatic classification of diseased and healthy regions is the missing link for total automation of most of the aforementioned approaches. Moreover, identification of regions could be used to guide and refine already existing tissue characterization algorithms, to ensure they only analyze plaque and not healthy regions, which are known to produce artifacts [8]. Therefore, an algorithm that can identify healthy and diseased vessel wall regions is a critical (and most of the time, missing) step and will have a tremendous benefit for true automation of OCT image processing.

**Fig. 1:**
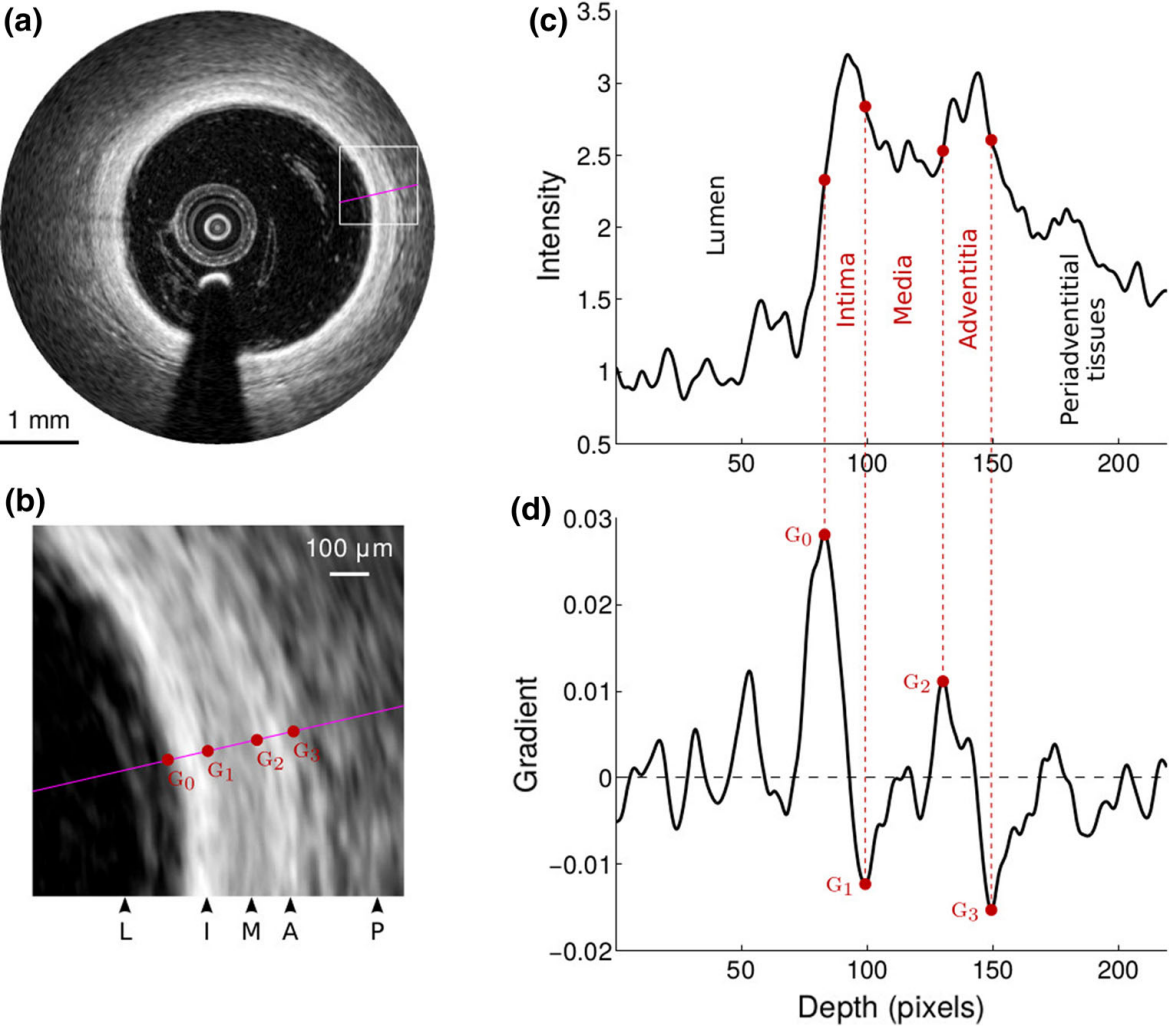
**a** OCT image of a human healthy coronary artery in vivo. The layered appearance of the wall is clearly visible. **b** Detail of the *square* region in (**a**), showing the lumen (L), the intima (I), media (M) and adventitia (A) layers, as well as the periadventitial tissues (P). **c** Intensity profile of the magenta line in (**a**, **b**). **d** Corresponding gradient. The extremum gradient values $\textsc {g}_{0,1,2,3}$ correspond to the LI, IM, MA and AP interfaces, respectively

A criterion for vessel health can be derived from the characteristics of the intima, media, and adventitia layers in the arterial wall (Fig. 1). To the best of our knowledge, no method has been introduced yet to segment the contours of the three arterial layers. In addition to classifying diseased and healthy regions, such analysis could be used to quantify the thickness of the anatomical tunicas and provide crucial information about the atherosclerosis process.

The main contributions of the proposed approach are the following. (1) The interfaces of the intima, media, and adventitia layers are simultaneously (as opposed to iteratively) extracted using an original contour segmentation method (“Multi-layer segmentation”). To address the challenge of multi-layers segmentation in two-dimensional (2D) cross-sectional images, a multi-parametric space is constructed in a higher-dimensional domain. (2) Healthy and diseased regions of the wall are identified (“Healthy region classification”). The tentative contours resulting from the segmentation operation are exploited by a classification method based on machine learning to detect healthy regions. (3) The method is fully automatic and does not require input from the user. (4) The framework is thoroughly validated on 260 previously unseen images, corresponding to 26 patients from two different medical centers (“Image analysis procedure”).

## Material and methods

In intracoronary OCT imaging, the triple-layered structure—intima, media, adventitia—can be seen in healthy arteries. On the other hand, in diseased arteries, the field of view is generally blocked by the presence of an atherosclerotic plaque or intima thickening, and most of the time only the intima layer is visible due to limited signal penetration. Based on this assumption, the framework consists of two fully automatic steps. First, a contour segmentation step is carried out in attempt to extract the contours of the intima, media, and adventitia layers. Second, the resulting tentative contours are exploited by a machine learning algorithm to classify healthy and diseased regions.

### Multi-layer segmentation

The purpose of the proposed segmentation method is to simultaneously extract the contours of the three anatomical interfaces, namely the intima–media (IM), media–adventitia (MA), and adventitia–periadventitia (AP) boundaries (Fig. 1a–c). This operation is performed along the entire circumference of the vessel wall, regardless of the state of the artery in the current image plane (*i.e.*, fully healthy, fully diseased, or partly healthy and diseased). The three contours are expected to be nearly parallel lines located in regions of extremum intensity gradient (*i.e.*, strongly positive or negative values, Fig. 1d). This a priori information is exploited by a dynamic programming approach to extract, in a 4D multi-parametric space, a unique and globally optimal solution that corresponds, in the 2D image plane, to three smooth paths that do not cross each other.

The present segmentation framework shares two aspects with a previous method proposed by our team [28]. Namely, the lumen-intima (LI) interface is segmented using the already existing method as a preprocessing step, and the initial cost map used for the front propagation is identical and defined as the gradient image. The essential aspects of the framework that define the originality of the present work are the following: (i) three anatomical interfaces (IM, MA, and AP) are targeted (as opposed to only two, namely the LI and abluminal plaque boundary), (ii) these boundaries are very close to each other and the IM and AP can easily be mistaken as they are located on ambiguous image regions with similar features, namely a strong negative gradient (as opposed to the LI and abluminal boundaries being unambiguous and located on positive and negative gradient regions, respectively), (iii) these three interfaces are extracted simultaneously (as opposed to iteratively), and (iv) a specific 4D search space is introduced (as opposed to 2D) to allow a unique front propagation scheme to localize the three target interfaces at once. All these points are further detailed below.

**Fig. 2:**
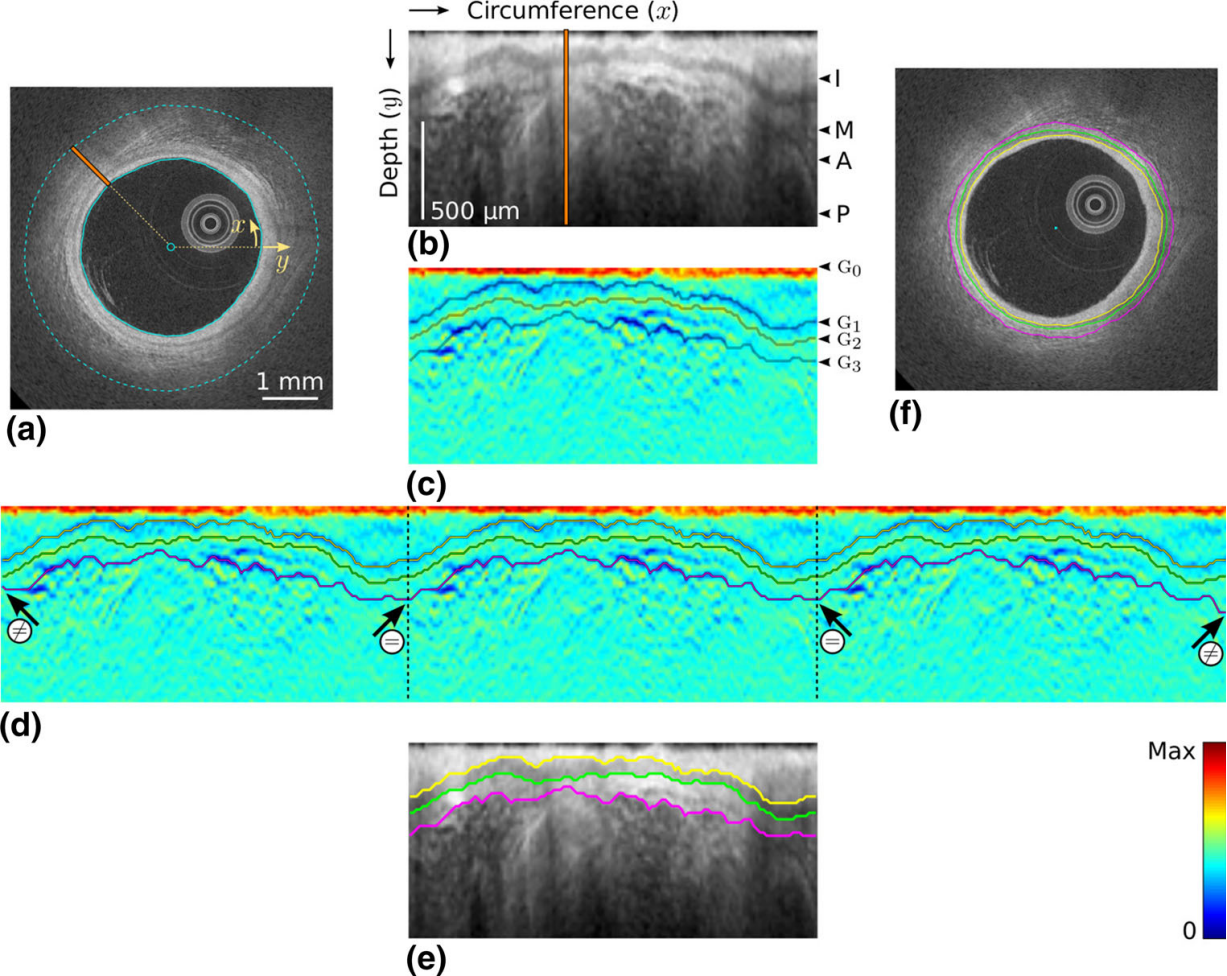
Principal steps of the segmentation process. In this example, the wall is entirely healthy and the anatomical layers are visible around the whole circumference. **a** Original image, with the lumen contour (*solid cyan line*). The depth $D=1~\text {mm}$ is indicated by the dashed cyan line. **b** Sub-image *I*, corresponding to the area between the two *cyan lines* in (**a**). The intima (I), media (M) and adventitia (A) layers, as well as the periadventitial tissues (P) are clearly visible. The *orange line* represents a single column, which corresponds to the *orange line* in (**a**). **c** Gradient image $I_G$. The IM, MA and AP interfaces, located in regions with extremum gradients values $\textsc {g}_{1,2,3}$, are delineated, although they are not determined yet at this stage of the process. **d** Replicated image $I'''_G$, with the IM (*yellow*), MA (*green*) and AP (*magenta*) contours resulting from the segmentation method. The contours within the central part of the image systematically respect the condition of continuity ($=$), even though the contours of the total image do not ($\ne$). **e** Segmentation result in the polar space, corresponding to the contours within the central part of $I'''_G$. **f** Final segmentation result in the Cartesian space

The segmentation framework is composed of a set of nine fully automatic operations (*i.e.*, no user interaction is required), as described hereafter.

(1): The LI interface is extracted using a previously introduced method [28].
(2): The region shadowed by the guidewire is masked out by detecting a continuous dark band in the averaged intensity profile of the pullback [25].
(3): A sub-image *I* is then generated from the original Cartesian image using the following approach (Fig. 2a, b). The entire circumference is first divided into a total of 100 angular steps originating from the lumen center. *I* is then generated by concatenating the 100 intensity profiles extracted from the luminal interface down to a depth $D=1~\text {mm}$ within the tissues.
(4): The expected location of the anatomical interfaces corresponds to regions with extremum gradient values (Fig. 1), namely strongly negative for the IM and AP interfaces, and strongly positive for the MA interface. The gradient image $I_G$ (Fig. 2c) is thus computed by applying a column-wise filter $G'_\sigma$ to the sub-image *I*:
  1 $$\begin{aligned} I_G = I *G'_\sigma \end{aligned}$$
  Here, $(*)$ is the convolution operator and $G'_\sigma$ is the first derivative of a Gaussian with standard deviation $\sigma$.
(5): The condition of contour continuity is then addressed: each IM, MA and AP contour must be closed in the Cartesian space (Fig. 2f). The issue is the following: since the image is processed in the polar space, the starting and ending points of a contour that runs from the left border of the image to the right are not guaranteed to have the same *y* coordinate if no specific rules are set. Therefore, when transforming such contour to the Cartesian space, there might be an abrupt discontinuity between the ending point of the contour (*i.e.*, $359^\circ$) and the starting point (*i.e.*, $0^\circ$). To enforce this condition, a periodic image $I'''_G$ is generated by replicating the gradient image $I_G$ three times along the *x* direction (Fig. 2d), such as:
  2 $$\begin{aligned} I'''_G = [I_G, I_G, I_G] \end{aligned}$$
(6): In the context of a front propagation scheme, two positive cost functions $\mathcal {C}^+$ and $\mathcal {C}^-$ are then built according to:
  3 $$\begin{aligned} \mathcal {C^\pm }=\mathcal {N}_{[0,1]}(\pm I'''_G) \end{aligned}$$
  where $N_{[0,1]}$ represents the normalization of a set of values to the positive interval [0, 1]. The points most likely to belong to the IM and AP interfaces therefore correspond to the regions with the lowest cost in the function $\mathcal {C}^+$. Similarly, the points most likely to belong to the MA interface correspond to the low-cost regions in $\mathcal {C}^-$.
(7): A dynamic programming scheme based on front propagation is then run to build a multi-parametric cumulative cost function $\mathbb {C}$. In this higher non-spatial dimensional domain, the space is described by a set of four parameters, and the following $\{x,y_1,y_2,y_3\}$ notation is adopted: *x* corresponds to the direction of the front propagation (*i.e.*, along the circumference of the vessel), and $y_{1,2,3}$ correspond to the depth of the IM, MA, and AP interfaces, respectively. The propagation is unidirectional along the *x* direction and favors low-cost regions in $\mathcal {C}^\pm$ while penalizing non-horizontal displacements. For $x=1$, $\mathbb {C}$ is initialized to zero. The function $\mathbb {C}$ is then iteratively built for increasing values of *x*, using the following relation:
  4 $$\begin{aligned} \begin{aligned} \mathbb {C}(x,y_1,y_2,y_3)&= \min _{dy_1,dy_2,dy_3} \\&\Big \{ \mathbb {C}(x-1,y_1\!+\!dy_1,y_2\!+\!dy_2,y_3\!+\!dy_3) \\&+\!~\omega _1 \cdot (1+\kappa \cdot dy_1) \\&\cdot \Big ( \mathcal {C}^{+}(x,y_1) \!+\! \mathcal {C}^{+}(x\!-\!1,y_1\!+\!dy_1) \Big ) \\&+\!~\omega _2 \cdot (1+\kappa \cdot dy_2) \\&\cdot \Big ( \mathcal {C}^{-}(x,y_2) + \mathcal {C}^{-}(x-1,y_2+dy_2) \Big ) \\&+~\omega _3 \cdot (1+\kappa \cdot dy_3) \\&\cdot \Big ( \mathcal {C}^{+}(x,y_3) \!+\! \mathcal {C}^{+}(x\!-\!1,y_3\!+\!dy_3) \Big ) \Big \} \end{aligned} \end{aligned}$$
  Here, the number of reachable neighbors along the *y* direction corresponds to $2N+1$, with $d_{y_1,y_2,y_3} \in \{ -N,\dots 0,\dots N \}$. The parameter $\kappa$ controls the smoothness of the contours, and the parameters $\omega _{1,2,3}$ control the weight that is set for each of the three interfaces. To ensure non-crossing contours that are separated from each other by a minimal gap *g*, the variables $y_{1,2,3}$ follow the relation $y_{n+1} \ge y_n + g$. A unique and global optimal path is then extracted from the multi-parametric cumulative cost function $\mathbb {C}$ using a previously described back tracking scheme [28]. This multi-parametric path *p* fully describes, for each column *x* of the sub-image *I*, the position $y_{1,2,3}$ of each of the three detected contours, such as:
  5 $$\begin{aligned} p(x)=\{y_1(x), y_2(x), y_3(x)\} \end{aligned}$$
(8): Since the extracted contours result from the segmentation of the replicated image $I'''_G$, they are truncated in three equal parts along the *x* dimension to match the width of the initial sub-image *I*. The two extreme parts are discarded, and the central part is kept, thus fulfilling the closed contours condition (Fig. 2e).
(9): Finally, the three 2D contours are mapped back in the original Cartesian coordinates of the cross-sectional OCT image to describe the location of the IM, MA and AP anatomical interfaces (Fig. 2f).

**Fig. 3:**
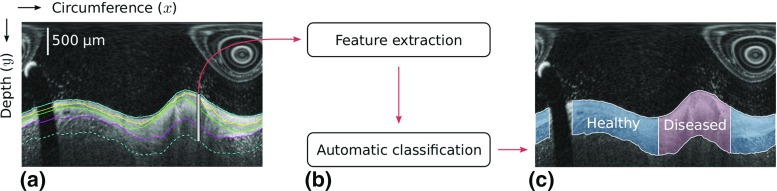
Schematic representation of the image classification. **a** The process is carried out column-wise in the sub-image *I*, in the region *R* (*vertical gray line*) defined by all the pixels between the luminal interface and the point located $300~\upmu \text {m}$ below the AP interface (*dashed line*). **b** Features are extracted from the region *R*. The corresponding column is then classified as healthy or diseased. **c** Resulting classification of the entire image

**Table 1:** Set of the 17 tentative features for image classification, sorted by decreasing relevance score

Score	Relevant	Feature
51/51	Yes	Contrast of the region *R* [9]
51/51	Yes	Mean square error between the intensity profile *I* of the region *R* and the linear interpolation of *R* as a polynomial of degree 1, calculated as $\text {Average}\big (|I(i)-P(i)|^2\big )$, where *P* is the 1-degree interpolation of *I*, and *i* is the index of the $i\mathrm{th}$ pixel along the A-line
50/51	Yes	Homogeneity of the region *R* [9]
50/51	Yes	Gradient $\textsc {g}_3$ at the MA interface
49/51	Yes	Correlation of the region *R* [9]
48/51	Yes	Gradient $\textsc {g}_2$ at the IM interface
34/51	Yes	Gradient $\textsc {g}_1$ at the LI interface
34/51	Yes	Monotony index, defined as the median absolute distance of all piece-wise permutations required to sort the intensity values of the region *R* in a monotonically decreasing fashion, calculated as $\text {Median}(|i-i'|)$, where $i'$ is the new index of *i* after the A-line has been sorted in a descending intensity order
22/51	No	Sum of all negative gradient values $I_G$ in the region *R*, calculated as $\sum _i\big (min(I_G(i), 0\big )$
21/51	No	Entropy of the region *R* [9]
13/51	No	Median image intensity in the media layer, between the IM and MA interfaces
12/51	No	Energy of the region *R* [9]
5/51	No	Median image intensity in the adventitia layer, between the MA and AP interfaces
4/51	No	Median image intensity in the intima layer, between the LI and IM interfaces
4/51	No	Distance between the LI and IM interfaces
3/51	No	Distance between the LI and AP interfaces
1/51	No	Distance between the LI and MA interfaces

### Healthy region classification

#### Rationale

In the context of machine learning, the contours resulting from the tentative segmentation of the three anatomical interfaces across the entire circumference of the artery are then exploited to extract a collection of image-based features. The aim is to automatically classify the arterial wall in healthy and diseased regions. The underlying rationale is that the resulting contours—and related image properties such as intensity and gradient values—are expected to have significantly different characteristics between these two types of regions, due to the presence (in healthy regions) or absence (in diseased regions) of the concentric layers that are targeted by the segmentation process, as illustrated in Fig. 3.

#### Feature selection

For each column of the sub-image *I*, a region *R* is defined by all the pixels between the top of the image (luminal interface) and the point located $300~\upmu \text {m}$ below the detected AP interface, as depicted in Fig. 3. To prepare a feature selection study, a set of 17 tentative features were extracted from each column of the region *R*, using the previously segmented contours, and subsequently normalized. These features are expected to yield substantially different values between a typical healthy wall profile and a diseased region (Fig. 1). Let us recall that the target pattern for healthy regions is a set of three layers bearing a high, low, and high image intensity, respectively. The extracted features are listed in Table 1.

To determine which features are important in the classification process, a feature selection study was performed using the R [15] package Boruta [13] algorithm, an heuristic procedure based on the random forest framework. The adopted protocol is the following. Among the 140 images of the training set, a subset of 100 A-lines was randomly gathered, together with the corresponding healthy or diseased label.^1^ Boruta was applied to identify the important features. The maximum number of iterations was 1000. The Bonferroni correction was applied. The value $p<0.01$ was considered to indicate a statistically significant difference. This experiment was conducted 51 times (odd number), each time with a different subset of randomly gathered A-lines from the training set. Finally, relevant features were defined as those that were labeled as “important” by Boruta at least 26 times (*i.e.*, for more than half of the 51 total number of experiments).

Results of the feature selection study were the following. Among the initial set of 17 features, a total of $N_\mathrm{F}=8$ feature were confirmed as relevant for the classification process, as described in Table 1. The remaining 9 features were confirmed as irrelevant. During the healthy wall detection phase, only the 8 relevant features were extracted from the image and provided to the classifier, and the 9 irrelevant features were ignored.

#### Classification

Classification was carried out with the adaptive boosting (AdaBoost) predictive algorithm [5]. In the present implementation, aiming to increase the robustness of the classification process against image noise and artifacts, four operations are subsequently carried out when analyzing the current $k\mathrm{th}$ frame, as described hereafter.

(1): For each column of the image, the $N_\mathrm{F}=8$ relevant features (Table 1) are extracted from the image and normalized.
(2): A similar operation is conducted in the $(k-1)\mathrm{st}$ and $(k+1)\mathrm{st}$ frames, namely features are computed in the two immediately adjacent frames of the pullback.
(3): The AdaBoost classifier is successively applied to the current $k\mathrm{th}$ image, as well as the two $(k-1)\mathrm{st}$ and $(k+1)\mathrm{st}$ images. For each column of the current $k\mathrm{th}$ frame, the resulting healthy or diseased label is determined by majority voting between the corresponding column in the three frames. The aim of this operation is to exploit the consistency of the data along the axis of the pullback to cope with frames that may be hindered by image noise.
(4): A set of morphological operations is applied to the healthy (1) and diseased (0) labels. The successive morphological operations consist in an erosion and a dilatation of kernel size $K_E$ and $K_D$, respectively. This final step contributes to correct some misclassified columns by aggregating them into larger homogeneous regions.

**Table 2:** Characteristics of the OCT scanners

	St Jude Medical Erasmus MC Rotterdam, The Netherlands	Terumo University Hospital of Auvergne Clermont-Ferrand, France
Pullback speed (mm/s)	20	20
Acquisition rate (fps)	100	158
Acquisition length (mm)	54	80
Number of frames	271	632
Resolution (axial/lateral) ($\upmu \text {m}$)	20/30	20/15
Depth of the scan range (mm)	4.3	4.5
Dimensions of the polar image (pixels)	968 $\times$ 504	512 $\times$ 512
Pixel size ($\upmu \text {m}$)	4.5	8.8

### Data collection

Forty OCT pullbacks, from 40 patients, were included in this study. To evaluate the performances of the framework on images acquired with different scanners and protocols, data were gathered from two different medical centers, namely the Thoraxcenter of Erasmus MC (Rotterdam, The Netherlands) and the University Hospital of Auvergne (Clermont-Ferrand, France). Twenty patients were included from both medical centers. All patients were suffering from coronary artery disease.

Pullbacks from Erasmus MC were acquired using the C7XR frequency-domain system and the Dragonfly intracoronary imaging catheter (Lightlab/St Jude Medical, Minneapolis, MN, USA), hereafter referred to as “SJM-OCT.” Pullbacks from University Hospital of Auvergne were acquired using the Lunawave coronary imaging console and the Fastview coronary imaging catheter (Terumo Corporation, Ashitaka, Japan), hereafter referred to as “Terumo-OCT.” The characteristics of both OCT scanners are provided in Table 2. All procedures followed were in accordance with the ethical standards of the responsible committee on human experimentation, and informed consent was acquired from all patients.

**Fig. 4:**
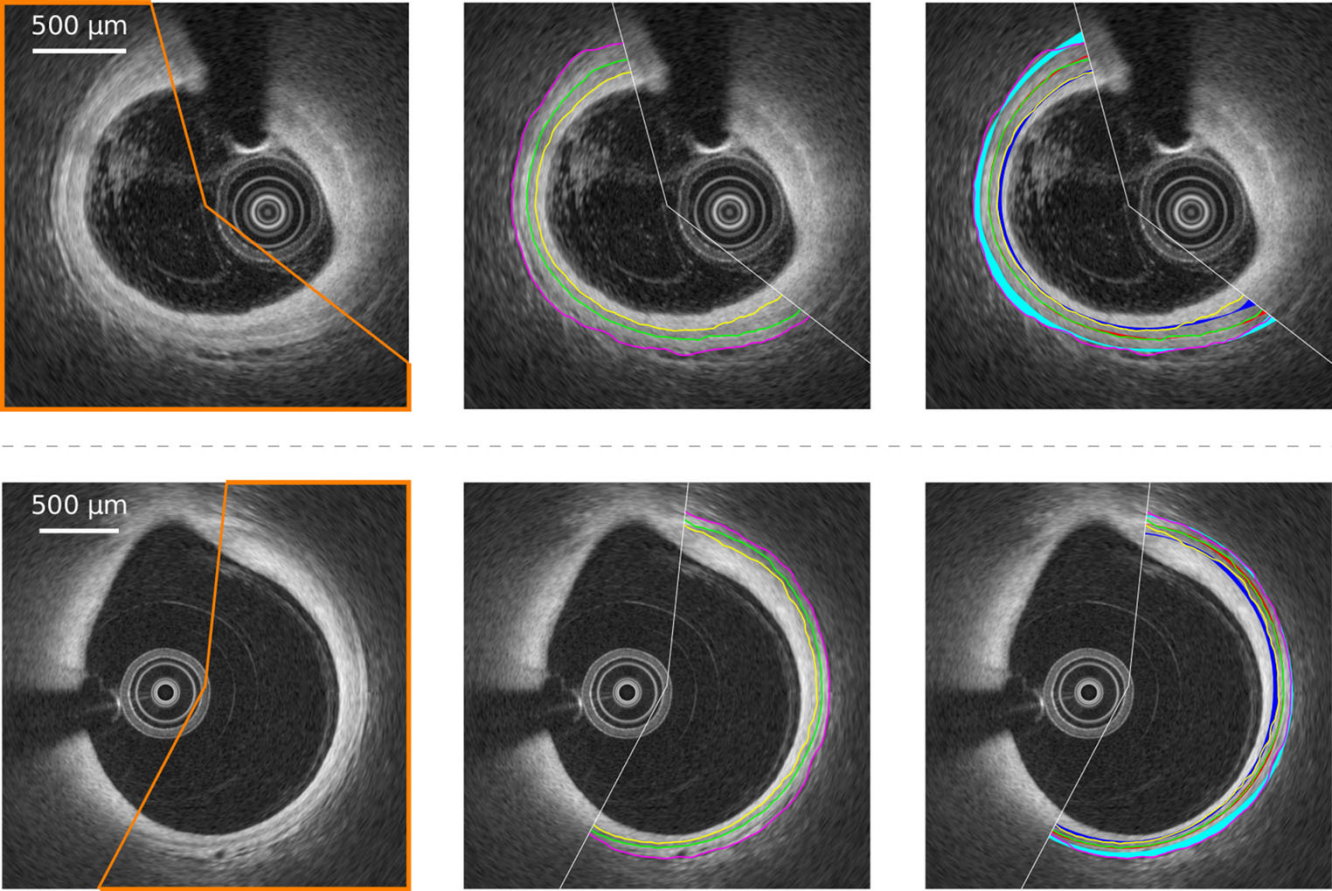
Representative examples of segmentation results, for SJM-OCT (*top row*) and Terumo-OCT (*bottom row*) patients. *Left column* the healthy wall region is indicated by the *orange line*. *Central column* the intima–media (*yellow*), media-adventitia (*green*), and adventitia–periadventitia (*magenta*) contours resulting from the method are represented. *Right column* the corresponding inter-analysts variability is indicated by the *blue*, *red* and *cyan* regions, for each angular step. The area representing the dispersion is centered around the mean position of the tracings of the analysts $\mathcal {A}_{1,2,3}$, and its width corresponds to one standard deviation in each direction around the mean value.

### Image analysis procedure

#### Image selection

For each pullback, 10 frames were selected for analysis, thus resulting in a total of 400 included images. To avoid subjective bias, the frame selection was random, with two criteria: absence of stent or bifurcation, and distance from each other of at least 10 intermediate frames.

#### Manual reference annotations

Reference annotations were manually traced on all the 400 included images. Inter-analyst variability was assessed by three experienced analysts $\mathcal {A}_1$  (GZ), $\mathcal {A}_2$  (AH), and $\mathcal {A}_3$  (NC), and intra-analyst variability was assessed by $\mathcal {A}_1$ performing the operations twice (denoted as $\mathcal {A}_1^{'}$). First, the region of interest encompassing the healthy tissues was indicated by an arc. Healthy wall regions were defined as regions where all three layers were visible, with an intima–media thickness inferior to $500~\upmu \text {m}$ [18]. Second, the three anatomical interfaces were precisely delineated, within the previously indicated arc region.^2^

#### Method evaluation

To evaluate both the segmentation and classification methods, a training set of 140 images was generated by randomly selecting 7 patients from both the SJM-OCT and Terumo-OCT cohorts. The training set was used during the development phase of the method, to empirically determine the optimal parameter settings and construct the AdaBoost model. A testing set of 260 images was then generated by selecting the remaining 13 patients from both cohorts. The testing set was used only during the evaluation phase of the method, to determine the performance of the developed framework on independent test data, using the previously determined optimal parameter settings and the previously generated AdaBoost model.

The segmentation accuracy was evaluated by means of a point-to-point distance comparison between the resulting contours and the manual tracings performed by the analyst $\mathcal {A}_1$. The classification accuracy was evaluated by comparing the automatic classification with the manual annotations performed by the analyst $\mathcal {A}_1$. The Dice coefficient [4], as well as an accuracy, sensitivity, and specificity analysis, were used to evaluate the similarity of the healthy arc regions between the manual annotations and the automatic method. The Dice coefficient *D* between two given regions *A* and *B* is an agreement metric that ranges between 0 (non-intersecting regions) and 1 (identical regions), and whose formula is given by:

6 $$\begin{aligned} D = \frac{2|A \cap B|}{|A|+|B|} \end{aligned}$$

Defining TP, TN, FP, FN as the number of true positives, true negatives, false positives and false negatives, respectively, the accuracy, sensitivity, and specificity were defined as:

7 $$\begin{aligned} \text {Accuracy}= & {} \frac{\mathrm{TP}+\mathrm{TN}}{\mathrm{TP}+\mathrm{TN}+\mathrm{FP}+\mathrm{FN}} \end{aligned}$$

8 $$\begin{aligned} \text {Sensitivity}= & {} \frac{\mathrm{TP}}{\mathrm{TP}+\mathrm{FN}} \end{aligned}$$

9 $$\begin{aligned} \text {Specificity}= & {} \frac{\mathrm{TN}}{\mathrm{TN}+\mathrm{FP}} \end{aligned}$$

## Results

### Parameter settings

For all parameters, the optimal setting was empirically determined using the training set ($n=140$), then the method was applied once to all the images of the testing set ($n=260$). The following configuration was used: standard deviation of the Gaussian function $G_\sigma$, $\sigma =30~\upmu \text {m}$; weight of the interfaces, $\omega _1 = 0.2$, $\omega _2 = 1$, and $\omega _3 = 1$; minimal gap between the contours, $g=45~\upmu \text {m}$; number of reachable neighbors, $2N+1=7$; smoothness coefficient, $\kappa =0.1$. The number of AdaBoost iterations was equal to 100. The size of the morphological kernels was $K_E=4$ and $K_D=9$, respectively.

### Contour segmentation

**Fig. 5:**
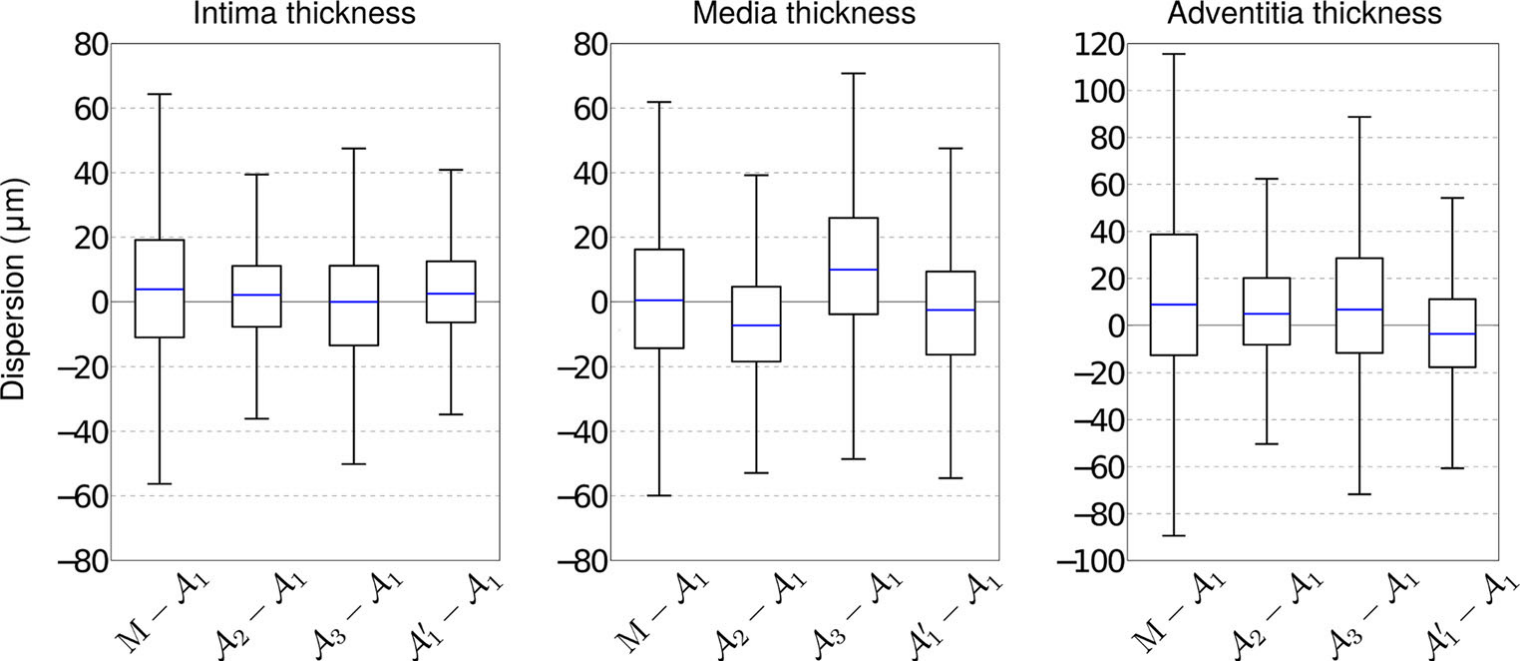
Box plot representing the dispersion between the layers thickness estimated with the proposed method *M* and the manual tracings performed by $\mathcal {A}_1$, compared to the inter- and intra-analysts variability, for the 26 pullbacks of the testing dataset. This corresponds to 10 images per pullback ($n=260$) for the method evaluation ($\text {M}-\mathcal {A}_1$), and 2 images per pullback ($n=52$) for inter- and intra-analysts variability. Percentiles are indicated by *boxes* ($25\mathrm{th}$ and $75\mathrm{th}$), inner lines ($50\mathrm{th}$), and error bars ($5\mathrm{th}$ and $95\mathrm{th}$)

The segmentation method was successfully applied to all the 400 images to extract the contours of the IM, MA, and AP interfaces. Representative results are provided in Fig. 4. The mean absolute errors (± standard deviation) of the contour segmentation method are displayed in Table 3. It is also relevant to describe the segmentation errors in relation to the thickness of the segmented layers: the mean absolute error to assess the intima–media thickness was $33 \pm 51~\upmu \text {m}$, with a bias of $+8~\upmu \text {m}$ and $95\%$ limits of agreement of $[-109, 125~\upmu \text {m}]$. For the 260 images used to assess the segmentation method, the average reference thickness of the layers was $131 \pm 67~\upmu \text {m}$ for the intima, $94 \pm 38~\upmu \text {m}$ for the media, and $103 \pm 64~\upmu \text {m}$ for the adventitia. For these images, the relative segmentation error, compared to the thickness of the corresponding layer, was $29 \pm 73~\%$ for the IM interface, $35 \pm 64~\%$ for the MA interface, $54 \pm 74~\%$ for the AP interface. When quantifying the thickness of the intima, media, and adventitia layers, the error introduced by the proposed method was very close to the inter- and intra-analyst variability, as displayed in Fig. 5.

**Table 3:** Segmentation absolute errors (mean ± SD) in $\upmu \text {m}$, for the proposed method and the manual tracings performed by the analysts $\mathcal {A}_{1,2,3}$

	Training set			Testing set		
	All	SJM-OCT	Terumo-OCT	All	SJM-OCT	Terumo-OCT
Intima–Media						
Method *vs* $\mathcal {A}_1$	25 ± 37	28 ± 44	23 ± 32	$\varvec{29 \pm 46}$	33 ± 48	27 ± 44
Inter-analysts	21 ± 25	21 ± 28	21 ± 22	20 ± 34	24 ± 45	18 ± 24
Intra-analyst	23 ± 40	31 ± 54	16 ± 20	15 ± 21	15 ± 18	15 ± 23
Media–adventitia						
Method *vs* $\mathcal {A}_1$	27 ± 42	29 ± 45	25 ± 39	$\varvec{30 \pm 50}$	31 ± 49	29 ± 50
Inter-analysts	20 ± 23	22 ± 28	19 ± 18	23 ± 48	27 ± 69	20 ± 25
Intra-analyst	20 ± 37	28 ± 52	14 ± 13	17 ± 28	20 ± 38	15 ± 20
Adventitia–tissues						
Method *vs* $\mathcal {A}_1$	37 ± 48	38 ± 49	37 ± 47	$\varvec{50 \pm 64}$	50 ± 66	49 ± 62
Inter-analysts	25 ± 28	27 ± 32	24 ± 25	32 ± 53	37 ± 72	29 ± 34
Intra-analyst	24 ± 40	34 ± 55	16 ± 20	24 ± 37	27 ± 47	22 ± 28

The metric is defined as the point-to-point distance along a line crossing the lumen center between a given contour and the corresponding reference contour. The number of included images per pullback for the method evaluation (Method *vs* $\mathcal {A}_1$) and for inter- and intra-analysts variability was 10 and 2, respectively

Bold values indicate the overall evaluation results of the method against the reference

### Healthy region classification

**Fig. 6:**
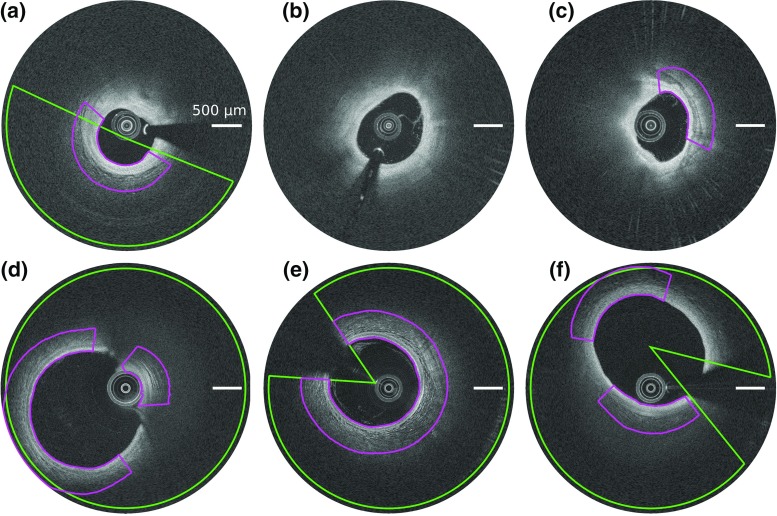
Examples of automatic healthy wall detection (*magenta*) compared to the manual tracings performed by $\mathcal {A}_1$ (*green*), for images of the testing set. The entire spectrum of results is represented. The panels (**a**–**c**) and (**d**–**f**) correspond to the SJM-OCT and Terumo-OCT cohorts, respectively. **a** The wall is partly healthy. **b**, **c** The entire circumference is diseased. **d**–**f** The entire circumference is healthy. **a**, **b**, **e** High agreement. **c** Failure, calcifications are wrongly detected by the method as this region has a layered appearance similar to a healthy wall structure. **d** Failure due to the tangential penetration angle of the probe, in contact with the arterial wall. **f** Failure, part of the healthy region is not detected by the method as the three anatomical layers are poorly visible

**Fig. 7:**
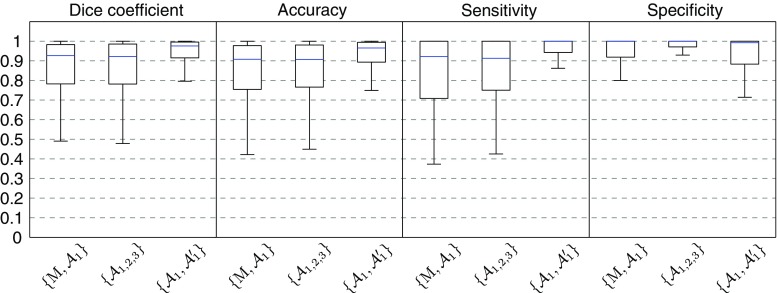
Box plot representing the Dice coefficient, accuracy, sensitivity, and specificity of healthy regions detection, between the proposed method M and the manual tracings performed by $\mathcal {A}_1$, compared to the inter- and intra-analysts variability, for all 260 images of the testing set. Percentiles are indicated by *boxes* ($25\mathrm{th}$ and $75\mathrm{th}$), *inner lines* ($50\mathrm{th}$), and *error bars* ($5\mathrm{th}$ and $95\mathrm{th}$)

**Fig. 8:**
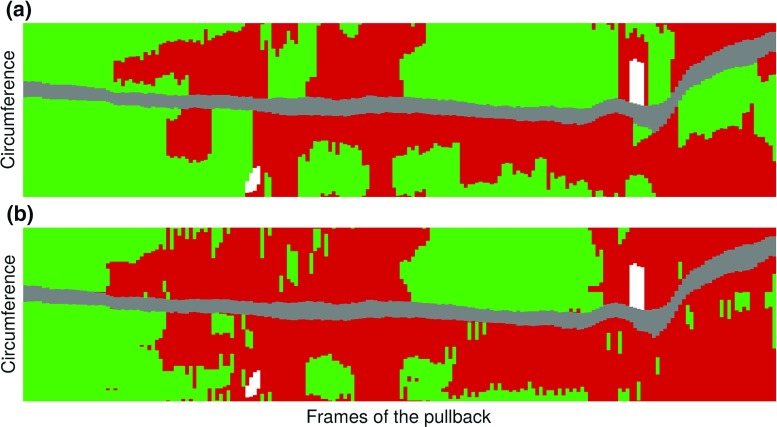
Example of the comparison of manual annotations (**a**) and automatic classification (**b**) in the first 200 frames of a pullback. Healthy regions, diseased regions, guidewire shadow, and sidebranches are represented in *green*, *red*, *gray*, and *white*, respectively. The Dice coefficient is equal to 0.80. The *x* and *y* axes represent the length of the arterial segment imaged during the pullback, and the angle from the lumen center, respectively

Subsequent to the contour extraction, the healthy region classification method was successfully applied to all the 260 images of the testing set to detect healthy regions of the wall from diseased regions. Representative classification results are displayed in Fig. 6. The healthy and diseased labels resulting from the automatic classification method were in good accordance with the manual annotations, as displayed in Fig. 7. For all the 260 analyzed images, the median value (and interquartile range) of the accuracy, sensitivity, and specificity was 0.91 ($[0.75 - 0.98]$), 0.92 ($[0.71 - 1.00]$), and 1.00 ($[0.92 - 1.00]$), respectively. The median Dice coefficient was 0.93, with an interquartile range of $[0.78 - 0.98]$ and an average (±SD) value of $0.83 \pm 0.25$. More specifically, the median, interquartile range, and average was 0.92, $[0.74 - 0.98]$, and $0.79 \pm 0.30$ for the SJM-OCT images, and 0.92, $[0.81 - 0.98]$, and $0.86 \pm 0.19$ for the Terumo-OCT images, respectively. For each of the 260 images of the testing set, the average percentage of healthy regions compared to the entire circumference of the wall, as annotated by the analyst $\mathcal {A}_1$, was $69 \pm 31\%$. This is to be compared with the corresponding ratio derived from the automatic method, which was $61 \pm 29\%$. To assess the usefulness of feature selection, these results were confronted to those obtained when using the full set of 17 features. Similar results were systematically found: the median value (and interquartile range) of the Dice coefficient, accuracy, sensitivity, and specificity were  0.92 ($[0.79 - 0.99]$), 0.89 ($[0.76 - 0.98]$), 0.93 ($[0.69 - 1.00]$), 1.00 ($[0.93 - 1.00]$), respectively. The healthy region classification method was finally applied to the first 200 frames of a single pullback as a proof of concept to automatically highlight healthy regions, as shown in Fig. 8.

### Computational speed

The present framework was implemented in MATLAB (MATLAB 7.14, The MathWorks Inc., Natick, MA, USA, 2011), on a desktop computer with 2.40 GHz processor with 12 Gb RAM. The average time required by the method to process one frame was 5 s to extract the contours of the 3 anatomical interfaces and 2 s to classify healthy and diseased regions. This is to be compared with the time required by manual annotations, corresponding to 180 s and 8 s, respectively.

## Discussion and conclusions

### On the contour segmentation method

Extraction of the anatomical interfaces is a critical step in the overall analysis, as the resulting contours are directly used to generate the classification features. Therefore, errors in the segmentation process would have a substantial impact in the subsequent classification process. Nevertheless, the performances of the segmentation method were validated and demonstrated an accuracy similar to the inter- and intra-analyst variability, as displayed in Table 3. Moreover, the segmentation errors were similar between the two cohorts, which contributes to demonstrate the robustness of the method. For both cohorts, the AP interface—generally less well anatomically defined—was more challenging to extract with accuracy, as demonstrated by the higher error rate. Nevertheless, accurate determination of the thickness of the intima–media complex is generally the most relevant clinical application.

When designing the front propagation scheme to simultaneously extract the three contours, the balance between low guidance (*i.e.*, image data only) and high guidance (*i.e.*, image data, and a number of geometrical a priori) is crucial. In the present framework, the resulting contours are subsequently exploited in the classification step to detect healthy and diseased regions. Therefore, a low guidance would result in irregular contours that are often erroneous in challenging image sections corrupted by noise, whereas a high guidance would result in contours that systematically respect a smooth and parallel structure and would compromise the classification process. In the present implementation, the guidance level is intermediate: first, a sub-image *I* is generated, where the LI interface is a horizontal line (Fig. 2), then the non-horizontal solutions are penalized by a smoothing term in the front propagation scheme (Eq. 4).

The problem of segmenting multiple layers was addressed in several studies, in the field of vascular OCT or intravascular ultrasound (IVUS), as well as retinal OCT as the structure of the human eye also exhibits several interfaces. These methods can be classified in two categories. The first category concerns the iterative extraction of the layers (*i.e.*, one after the other) that cannot perform a simultaneous segmentation of all contours. A combination of region-growing and clustering was used to segment the LI and MA interfaces with a two-step approach [6]. A Viterbi-based dynamic programming implementation was proposed to iteratively segment multiple layers, where the final solutions are obtained by uncrossing all the extracted contours [14]. A fast-marching method was presented to segment the LI and MA contours [3], although a drawback of this approach is the initialization procedure, since two initial contours must be placed around each interface to be segmented. A coarse-to-fine approach, based on a combination of active contours and Markov random fields, and following three successive steps where the large structures are extracted first and the fine structures last, was also introduced [7]. A method based on support vector machine was proposed to segment the LI and MA interfaces, using a set of features based on texture, geometry, edge presence, and shadow [2]. The second category includes methods devised to simultaneously extract multiple contours, similarly to the approach presented in this manuscript. Six intra-retinal layers were segmented using a method based on multi-phase active contours [27]. Nevertheless, this approach is hindered by two limitations: it requires a manual initialization (three points per contour), and convergence relies on multiple iterations that are not always guaranteed to yield the global optimal result. Graph optimization was exploited under the min-flow theory to simultaneously segment the LI and MA interfaces [17], as well as seven retinal layers (that is, 3 layers were first simultaneously segmented, then the remaining 4 also simultaneously) in ophthalmic OCT [16]. The segmentation method proposed here is comparable to these two latter approaches [16, 17], in the sense that a combinatorial analysis is performed to extract the optimal solution corresponding to the simultaneous delineation of several contours, the difference being that the algorithm adopted in the present framework is based on front propagation rather than graph cut.

The accuracy of the segmentation framework may be penalized by the presence of artifacts. The method is not devised to detect the presence of stents and extract their contours and is expected to fail in such cases. Future perspectives will focus on a cascading approach, where stented regions are first detected using a specifically devised method [10, 12, 19], before the tissues in the remaining of the pullback are segmented using the present approach. Thrombi were not present in our dataset, and such images were therefore not evaluated; however, they are expected to yield erroneous contours with irregular shapes (from our experience, we also expect the classification method to provide correct results, as the features that will be derived from the contours would be substantially different from those of the healthy wall structure). It is, however, noteworthy that the method performed well when flushing was not optimal.

### On the healthy region classification method

The main originality of the proposed classification method is to directly exploit the tentative contours resulting from the previous segmentation step to partition the image in healthy and diseased regions. A collection of local features is extracted from specific image regions delimited by the contours (*e.g*, image statistics at the contours location). This approach is different from previous studies that used a unique and more global feature such as the backscattering coefficient [26] or the attenuation coefficient [21].

A limitation of the proposed approach is its dependency to a clear layered architecture, with visible intima, media, and adventitia tunicas. Tangential penetration is the artifact that most hinders the method: in case of an eccentric catheter, the sharp angle of incidence of the beam would result in an image where part of the healthy layers are not clearly defined [22]. In such a situation, the classification algorithm may provide inaccurate results, as illustrated in Fig. 6d. The healthy region classification process also failed in some cases where all three human analysts correctly annotated the region. This misclassification concerned two types of failures: false negatives and false positives. False negatives corresponded to healthy images that could not be correctly identified when the structure was not well defined, with blurred and poorly perceptible layers, as depicted in Fig. 6f. False positives corresponded to diseased regions that were wrongly labeled as healthy because of image artifacts or plaque formation. Such cases include fibrotic plaques with a layer-like structure, or calcific plaques showing a well contrasted pattern, as shown Fig. 6c). Misclassification issues could be solved in a future implementation of the AdaBoost model, by specifically integrating a large number of such challenging cases in the learning process. However, it is noteworthy that false positives were less frequent than false negatives. This is represented in Fig. 7 with the specificity being substantially higher than the sensitivity. From a clinical perspective, this would have the following implication: the classification results for regions labeled as healthy are trustworthy, and only regions labeled as diseased—that must be analyzed anyway—should be visually inspected to confirm the classification result. Let us also note that the accuracy (Dice coefficient, accuracy, sensitivity and specificity) is very close to the inter-observer variability (Fig. 7), which contributes to validate the performances of the method.

The proposed classification scheme is binary, namely a healthy or diseased label is applied to different regions of the image. In contrast to several previous studies [1, 20, 21, 24, 26], the present method is not capable of characterizing different tissue types, such as lipid, fibrous, or calcium. To address this limitation, future work will focus on a cascade approach, where the artery is first roughly partitioned in healthy and diseased regions using the present framework, before a second classifier based on different image features is applied only to diseased regions for finer tissue and plaque type characterization. However, the present method can potentially be used as a preprocessing step to guide existing classification approaches in diseased regions.

### Conclusions

A contour segmentation method based on dynamic programming was introduced to localize the contours of the intima, media, and adventitia layers in intracoronary OCT images. A unique and globally optimal multi-parametric path was extracted to describe the set of three 2D contours. The tentative contours resulting from the segmentation method were then exploited by a machine learning algorithm to partition the images in healthy and diseased regions. To thoroughly evaluate the method and assess its clinical applicability potential, the framework was trained on 140 images and validated on 260 other images, gathered from two different medical centers with two different OCT scanners. The clinical significance of the present study is twofold. First, segmenting the arterial layers in healthy regions enables accurate and automatic quantification the intima–media thickness, one of the most relevant clinical parameters. Second, fully automatic identification of the diseased regions provides valuable information for the clinician because it indicates the regions that must be further analyzed. Such an approach, allowing a distinction of healthy and diseased regions, is an essential step for plaque characterization. In conclusion, the introduced framework has potential to assist clinicians with quantified information in an automatic, accurate, and reproducible fashion.

## Footnotes

1 We kindly ask the reader to refer to “Image analysis procedure” section for a full description of the study design regarding the generation of the training set as well as the manual labels.

2 Due to the highly time-consuming nature of manual contour tracing, inter- and intra-analyst variability was only assessed on a subset of 80 images. This subset was generated by randomly selecting, for each pullback, 2 frames among the 10 initial frames.
